# Nonadherence to Multimodality Cancer Treatment Guidelines in the United States

**DOI:** 10.1016/j.adro.2022.100938

**Published:** 2022-03-08

**Authors:** Leila T. Tchelebi, Biyi Shen, Ming Wang, Louis Potters, Joseph Herman, Daniel Boffa, Joel E. Segel, Henry S. Park, Nicholas G. Zaorsky

**Affiliations:** a Department of Radiation Medicine, Zucker School of Medicine, Hempstead, New York; b Department of Radiation Medicine, Northwell Health Cancer Institute, Mount Kisco, New York; c Department of Public Health Sciences, Penn State College of Medicine, Hershey, Pennsylvania; d Department of Surgery, Yale School of Medicine, New Haven, Connecticut; e Department of Health Policy Administration, Penn State University, University Park, Pennsylvania; f Department of Therapeutic Radiology, Yale School of Medicine, New Haven, Connecticut; g Department of Radiation Oncology, University Hospitals Seidman Cancer Center, Case Western Reserve School of Medicine, Cleveland, Ohio

## Abstract

**Purpose:**

Our purpose was to identify patients with cancer who do not receive guideline-concordant multimodality treatment and to identify factors that are associated with nonreceipt of guideline-concordant multimodality treatment.

**Methods and Materials:**

Five cancers for which the multimodal guideline-concordant treatment (with surgery, chemotherapy, and radiation therapy) is clearly defined in national guidelines were selected from the National Cancer Database: (1) nonmetastatic anal cancer, (2) locally advanced cervical cancer, (3) nonmetastatic nasopharynx cancer, (4) locally advanced rectal cancer, and (5) locally advanced non-small cell lung cancer. Multivariable logistic regression was used to determine the odds ratios (with 95% confidence intervals) of receiving the guideline-concordant treatment versus not, adjusting for common confounding variables.

**Results:**

178,005 patients with cancer were included: 32,214 anal, 54,485 rectal, 13,179 cervical, 5061 nasopharyngeal, and 73,066 lung. Overall, 162,514 (91%) received guideline-concordant treatment and 15,491 (9%) did not. Twenty-one percent of patients with cervical cancer, 10% of patients with rectal cancer, 7% of patients with lung cancer, 5% of patients with anal cancer, and 3% of patients with nasopharynx cancer did not receive guideline-concordant treatment. In general, patients who were older, with comorbid conditions, and who were evaluated at low-volume facilities (odds ratios > 1 with *P* < .05) were less likely to receive guideline-concordant treatment.

**Conclusions:**

Nearly 1 in 10 patients in this cohort are not receiving appropriate multimodal cancer therapy. There appear to be significant disparities in receipt of guideline-concordant treatment based on primary tumor site, age, comorbidities, and reporting facility.

## Introduction

Each year, approximately 1.7 million people are diagnosed with cancer in the United States and treated at thousands of facilities located across the country. In 1999, the Institute of Medicine published a report indicating that many patients with cancer were not receiving the care known to be effective for their disease based on the best available evidence.^1^ This report precipitated an increased demand for the implementation of national guidelines, such as those issued by the National Comprehensive Cancer Network (NCCN), to standardize cancer care across the country.^2^, ^3^, ^4^

The NCCN guidelines are based on clinical trials that have undergone a rigorous peer-review process to ensure the safety and efficacy of the treatments proposed. The NCCN guidelines contain consensus recommendations for clinical scenarios based on available evidence, including randomized and nonrandomized data. The goal of the NCCN guidelines is not only to ensure uniformity of cancer care across the United States but also to ensure that patients are receiving the best available treatment for their disease. Indeed, studies have shown that adherence to treatment guidelines results in improved survival for patients.^5^, ^6^, ^7^, ^8^, ^9^

The purpose of the present work was to evaluate factors associated with receipt of care consistent with the evidence-based guidelines outlined by the NCCN across a variety of cancer sites for which multimodal therapy is recommended. We specifically focused on cancers for which there has been uniform NCCN consensus regarding the use of surgery, chemotherapy, and radiation therapy over the period of study. We hypothesized that there is a substantial proportion of patients with cancer who are not receiving guideline-concordant treatment and that nonadherence to guideline-concordant treatment is associated with patient and facility factors.

## Methods and Materials

### Data extraction and synthesis

The National Cancer Database (NCDB) is a hospital-based cancer registry that collects data from American College of Surgeons–Commission on Cancer accredited facilities. It is the largest cancer registry worldwide, including 70% of all malignant cancers diagnosed in the United States from more than 1400 hospitals accredited by the Commission on Cancer.^10^ Centers included range from small community hospitals to large academic medical centers and National Cancer Institute-designated comprehensive cancer centers.^11^ The NCDB records patient demographics, socioeconomic characteristics, comorbidities, tumor characteristics, facility characteristics, information regarding therapies delivered, and survival data.^12^

### Patients

The NCDB was queried for patients with cancers for which the recommendations regarding management are clearly defined in the NCCN guidelines. We specifically selected cancers with clinical scenarios (both site and stage) for which there is only one proposed treatment approach within the NCCN guidelines, without additional branch points or multiple acceptable treatment options. Treatment had to include multimodal therapy. The most recent NCCN guidelines for each cancer site were used to define guideline-concordant treatment.^13^, ^14^, ^15^, ^16^, ^17^ Sites included were anus, nasopharynx, cervix, rectum, and non-small cell lung. Included patients had the most common histologies for each site: squamous cell for anus, nasopharynx, and cervix; adenocarcinoma for rectum; and a mix of squamous cell (55%) and adenocarcinoma (45%) for lung. Patients were included from 2004 to 2015, except for rectal cancer. Patients with rectal cancer were only included after 2006 because the seminal paper establishing the current guideline-concordant treatment for rectal cancer was published in 2004,^18^ and we wanted to allow time for centers to adopt the new treatment paradigm. We reviewed previous versions of the NCCN guidelines to ensure that the recommendations were consistent over the study period. Detailed information regarding inclusion and exclusion criteria for each cancer site is presented in Supplementary Figures 1-5. As far as stage, although the current guidelines are based on the 8th edition of the American Joint Committee on Cancer Staging Manual, versions 6 and 7 would have been used over the study period. To the best of our ability, we verified that the overall stage groupings did not change over the study period, given that the overall stage groupings are what dictate NCCN treatment recommendations.

### Definition of standard of care

We used definitive local therapy (surgery or radiation) to define guideline-concordant treatment to exclude patients who received no treatment or received palliative systemic therapy alone due to inability to tolerate definitive local therapy, as this may not necessarily have been inappropriate. Table E1 shows the definitions of guideline-concordant treatment versus nonguideline-concordant treatment, as well as the recommended radiation dose, chemotherapy regimens, and data in support of these recommendations.

### Statistical analysis

The data are presented as means and standard deviations for continuous variables and frequencies with percentage (%) for categorical variables. Multivariable logistic regression was used to estimate the odds ratio (OR) of receiving guideline-concordant treatment versus not by cancer site, adjusting for a number of covariates. These included age (in years, as a continuous variable), distance to treatment facility (in miles, as a continuous variable), race (white, black, other), Charlson-Deyo comorbidity score levels (0, indicating no comorbid conditions; 1, indicating a comorbidity score of 1; ≥2, indicating a comorbidity score of 2 or more), income (<$38,000, $38,000-$47,999, $48,000-$62,999, ≥$63,000), insurance status (none, private, Medicaid, Medicare, other), facility type (community cancer program, comprehensive community cancer program, academic/research program, integrated network cancer program), treatment facility location (metro, urban, rural), number of treating facilities (all treatment received at 1 vs more than 1 facility), and facility volume (low- and high-volume reporting facility as determined by the median facility volume). We did not include sex because cervical cancer is sex-specific. All statistical analyses were performed with R, version 3.5.1, with 2-sided tests and statistical significance level of α = 0.05.

## Results

**Fig. 1:**
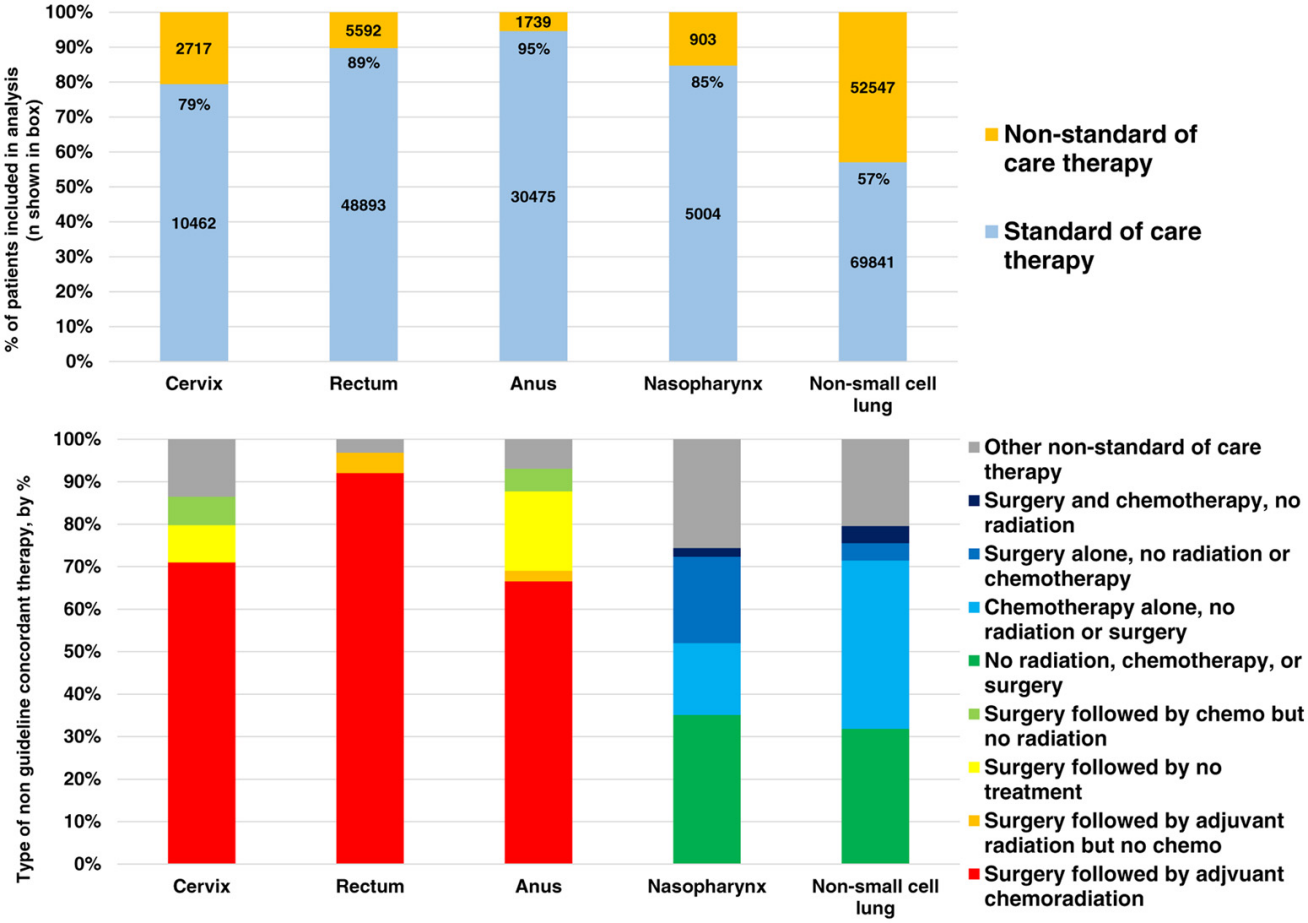
Treatments delivered to patients. (A) Percent of patients receiving nonguideline-concordant therapy versus guideline-concordant therapy by disease site. (B) Percent of patients receiving specific nonguideline concordant treatments by disease site.

Of the 178,005 patients included in our analysis, there were 32,214 with anal, 54,485 with rectal, 13,179 with cervical, 5061 with nasopharyngeal, and 73,066 with non-small cell lung cancer (NSCLC). Most patients were white, with no comorbidities, and were insured. Patient characteristics are listed in Table 1. Overall, 162,514 (91%) received guideline-concordant treatment and 15,491 (9%) did not, although this varied by cancer site. As shown in Fig 1, 5300 (7%) patients with NSCLC, 2717 (21%) patients with cervical, 5592 (10%) patients with rectal, 143 (2.8%) patients with nasopharyngeal, and 1739 (5%) patients with anal cancer did not receive guideline-concordant treatment.

**Table 1:** Characteristics of patients receiving guideline-concordant treatment versus nonguideline-concordant treatment, by cancer site

		Anal		Rectal		Cervix		Lung		Nasopharynx	
Variable		GCT n (%)	Non-GCT n (%)	GCT n (%)	Non- GCT n (%)	GCT n (%)	Non- GCT n (%)	GCT n (%)	Non- GCT n (%)	GCT n (%)	Non- GCT n (%)
Race	White	26,667 (87.5)	1469 (84.5)	42,295 (86.5)	4807 (86.0)	7835 (74.9)	2142 (78.8)	56,598 (83.5)	4630 (87.4)	3093 (62.9)	108 (75.5)
	Black	3052 (10.0)	213 (12.2)	3816 (7.8)	496 (8.9)	1781 (17.0)	375 (13.8)	8993 (13.3)	469 (8.8)	640 (13.0)	21 (14.7)
	Other	756 (2.5)	57 (3.3)	2782 (5.7)	289 (5.2)	846 (8.1)	200 (7.4)	2175 (3.2)	201 (3.8)	1185 (24.1)	14 (9.8)
Charlson comorbidity score	0	24,651 (80.9)	1321 (76.0)	38,913 (79.6)	4344 (77.7)	9024 (86.3)	2335 (85.9)	42,921 (63.3)	3182 (60.0)	4222 (85.8)	119 (83.2)
	1	3765 (12.3)	287 (16.5)	7933 (16.2)	991 (17.7)	1186 (11.3)	332 (12.2)	17,561 (25.9)	1546 (29.2)	542 (11.0)	20 (14.0)
	≥2	855 (2.8)	69 (4)	1517 (3.1)	188 (3.4)	185 (1.8)	37 (1.4)	5512 (8.1)	465 (8.8)	117 (2.4)	4 (2.8)
Income	<$38,000	5741 (18.8)	341 (19.6)	8289 (16.9)	1021 (18.3)	2654 (25.4)	568 (20.9)	15,204 (22.4)	932 (17.6)	898 (18.3)	23 (16.1)
	≥$38,000 and <$48,000	7444 (24.4)	457 (26.3)	11,882 (24.3)	1373 (24.6)	2817 (26.9)	677 (24.9)	17,425 (25.7)	1294 (24.4)	1122 (22.8)	35 (24.5)
	≥$48,000 and <$63,000	8078 (26.5)	450 (25.9)	13,237 (27.1)	1495 (26.7)	2592 (24.8)	728 (26.8)	17,700 (26.1)	1384 (26.1)	1268 (25.8)	38 (26.6)
	≥$63,000	8995 (29.5)	472 (27.1)	15,172 (31.0)	1667 (29.8)	2280 (21.8)	712 (26.2)	16,082 (23.7)	1584 (29.9)	1589 (32.3)	46 (32.2)
Insurance	Uninsured	1618 (5.3)	86 (5.0)	2250 (4.6)	268 (4.8)	1190 (11.4)	204 (7.5)	2758 (4.1)	153 (2.9)	245 (5.0)	3 (2.1)
	Private	13,585 (44.6)	654 (37.6)	24,656 (50.4)	2605 (46.6)	4199 (40.1)	1402 (51.6)	20,264 (29.9)	2090 (39.4)	2598 (52.8)	85 (59.4)
	Medicaid	2798 (9.2)	174 (10.0)	3513 (7.2)	365 (6.5)	2899 (27.7)	528 (19.4)	5258 (7.8)	336 (6.3)	572 (11.6)	16 (11.2)
	Medicare	11,477 (37.7)	772 (44.4)	16,853 (34.5)	2159 (38.6)	1879 (18.0)	454 (16.7)	36,870 (54.4)	2568 (48.5)	1328 (27.0)	31 (21.7)
	Other government	519 (1.7)	22 (1.3)	715 (1.5)	105 (1.9)	138 (1.3)	32 (1.2)	1459 (2.1)	66 (1.2)	98 (2.0)	0 (0)
Facility type	Academic research	9563 (31.4)	675 (38.8)	17,352 (35.5)	1653 (29.6)	4438 (42.4)	843 (31.0)	20,011 (29.5)	1991 (37.6)	1837 (37.4)	76 (53.1)
	Community cancer program	3200 (10.5)	152 (8.7)	4004 (8.2)	649 (11.6)	445 (4.2)	125 (4.6)	8702 (12.8)	449 (8.5)	356 (7.2)	8 (5.6)
	Comprehensive community cancer program	13,657 (44.8)	696 (40.0)	19,962 (40.8)	2553 (45.6)	2715 (24.9)	871 (32.1)	31,683 (46.8)	2254 (42.5)	1679 (34.1)	37 (25.9)
	Integrated network cancer program	3134 (10.3)	163 (9.4)	5190 (10.6)	508 (9.1)	968 (9.2)	243 (8.9)	6984 (10.3)	540 (10.2)	491 (10.0)	5 (3.5)
Facility number	All treatment at 1 CoC facility	27,212 (89.3)	1561 (89.8)	35,489 (72.6)	4364 (78.0)	7731 (73.9)	1923 (70.8)	56,592 (84)	3836 (72.4)	4046 (82.3)	116 (81.1)
	Treatment at > 1 CoC facility	3263 (10.7)	178 (10.2)	13,404 (27.4)	1228 (22.0)	2731 (26.1)	794 (29.2)	10,814 (16)	1464 (27.6)	872 (17.7)	121 (13.4)
Facility volume	Low	5856 (19.2)	328 (18.9)	7477 (15.3)	1224 (21.9)	597 (5.7)	252 (9.3)	14,367 (21.2)	924 (17.4)	846 (17.2)	22 (15.4)
	High	24,619 (80.8)	1411 (81.1)	41,416 (84.7)	4368 (78.1)	9865 (94.3)	2465 (90.7)	53,399 (78.8)	4376 (82.6)	4072 (82.8)	121 (84.6)
Facility location	Metro	25,098 (82.4)	1350 (77.6)	38,300 (78.3)	4438 (79.4)	8407 (80.4)	2196 (80.8)	52,015 (76.8)	4175 (78.8)	4109 (83.5)	121 (84.6)
	Urban	4105 (13.5)	290 (16.7)	8170 (16.7)	902 (16.1)	1555 (14.9)	386 (14.2)	11,692 (17.2)	793 (15.0)	582 (11.8)	18 (12.6)
	Rural	492 (1.6)	43 (2.5)	1197 (2.5)	115 (2.1)	161 (1.5)	47 (1.7)	1611 (2.4)	111 (2.1)	74 (1.5)	2 (1.4)
Total	N/A	30,475 (95)	1739 (5)	48,893 (90)	5592 (10)	10,462 (79)	2717 (21)	67,766 (92.7)	5300 (7.3)	4918 (97.2)	143 (2.8)

*Abbreviations:* CI = confidence interval; CoC = commission on cancer; GCT = guideline-concordant therapy; K = thousand, N/A = not applicable; OR = odds ratio.

Factors associated with nonadherence to guideline-concordant treatment varied widely among cancer subtypes (Table 2). For patients with anal cancer, older age (OR, 1.01; 95% confidence interval [CI], 1.01-1.02; *P* < .0001), black race (OR, 1.25; 95% CI, 1.04-1.48; *P* = .0001), other nonwhites (OR, 1.40; 95% CI, 1.04-1.85; *P* = .02) with 1 (OR, 1.33; 95% CI, 1.16-1.53; *P* < .0001) or Charlson-Deyo comorbidity scores of 2 or more (OR, 1.33; 95% CI, 1.01-1.72; *P* = .04), who received treatment in an urban (OR, 1.39; 95% CI, 1.20-1.61; *P* < .0001) or rural facility (OR, 1.83; 95% CI, 1.29-2.52; *P* < .0001) were more likely to receive nonguideline-concordant treatment. Meanwhile, treatment at a nonacademic facility or at a high-volume center (OR, 0.83; 95% CI, 0.71-0.98; *P* = .03) was associated with decreased likelihood of nonadherence.

**Table 2:** Results from multivariable logistic regression (nonadherence to guideline-concordant treatment versus adherence to guideline-concordant treatment) by cancer site

		Anal	Rectal	Cervix	Lung	Nasopharynx
Variable		OR (95% CI)	OR (95% CI)	OR (95% CI)	OR (95% CI)	OR (95% CI)
Age (in years, 1 unit increase)	N/A	1.01 (1.01-1.02)	1.01 (1.01-1.02)	0.99 (0.99-1.00)	0.98 (0.98-0.98)	1.00 (0.98-1.03)
Distance to treatment facility (in miles, 100-unit increase)	N/A	1.05 (1.01-1.09)	0.97 (0.89-1.05)	1.07 (1.00-1.14)	1.08 (1.05-1.10)	1.00 (0.99-1.00)
Race	White	Reference	Reference	Reference	Reference	Reference
	Black	1.25* (1.04-1.48)	1.12* (1.01-1.25)	0.91 (0.78-1.05)	0.64* (0.57-0.71)	0.82 (0.43-1.45)
	Other	1.40* (1.04-1.85)	0.95 (0.83-1.08)	0.89 (0.73-1.08)	1.03 (0.87-1.21)	0.30* (0.15-0.55)
Charlson comorbidity score	0	Reference	Reference	Reference	Reference	Reference
	1	1.33* (1.16-1.53)	1.05 (0.98-1.14)	1.22* (1.06-1.41)	1.32* (1.24-1.41)	1.41 (0.81-2.34)
	2	1.33* (1.01-1.72)	1.02 (0.87-1.19)	0.81 (0.54-1.17)	1.34* (1.21-1.49)	1.43 (0.42-3.59)
Income	<$38,000	Reference	Reference	Reference	Reference	Reference
	≥$38,000 and <$48,000	1.11 (0.95-1.30)	0.94 (0.85-1.03)	0.96 (0.83-1.11)	1.13* (1.03-1.23)	1.79 (0.96-3.46)
	≥$48,000 and <$63,000	1.07 (0.91-1.26)	0.91 (0.83-1.00)	1.11 (0.95-1.29)	1.14* (1.04-1.25)	1.58 (0.85-3.09)
	≥$63,000	1.03 (0.88-1.23)	0.90* (0.82-0.99)	1.20* (1.02-1.40)	1.34* (1.22-1.48)	1.36 (0.72-2.66)
Insurance	Uninsured	Reference	Reference	Reference	Reference	Reference
	Private	0.89 (0.70-1.15)	0.95 (0.82-1.10)	1.88* (1.55-2.30)	1.87* (1.57-2.26)	3.14 (0.97-19.28)
	Medicaid	1.16 (0.87-1.54)	0.87 (0.73-1.04)	1.10 (0.89-1.37)	1.20 (0.98-1.49)	2.91 (0.79-18.76)
	Medicare	1.04 (0.81-1.40)	0.91 (0.78-1.07)	1.62 (1.29-2.06)	1.67* (1.39-2.03)	1.97 (0.55-12.61)
	Other government	0.66 (0.38-1.08)	1.32* (1.03-1.70)	1.02 (0.58-1.70)	0.86 (0.63-1.18)	0.00 (0.00-1.65)
Facility type	Academic research	Reference	Reference	Reference	Reference	Reference
	Community cancer program	0.53* (0.42-0.66)	1.27* (1.12-1.43)	1.24 (0.96-1.59)	0.55* (0.48-0.63)	0.49 (0.19-1.12)
	Comprehensive community cancer program	0.69* (0.61-0.77)	1.23* (1.14-1.32)	1.61* (1.43-1.80)	0.73* (0.69-0.79)	0.49* (0.31-0.75)
	Integrated network cancer program	0.72* (0.59-0.86)	0.94 (0.84-1.05)	1.30* (1.10-1.54)	0.75* (0.67-0.83)	0.23* (0.08-0.53)
Facility number	All treatment at 1 CoC facility	Reference	Reference	Reference	Reference	Reference
	Treatment at >1 CoC facility	0.96 (0.81-1.14)	0.74* (0.69-0.79)	1.04 (0.92-1.16)	1.96* (1.83-2.09)	0.97 (0.59-1.55)
Facility volume	Low	Reference	Reference	Reference	Reference	Reference
	High	0.83* (0.71-0.98)	0.70* (0.64-0.77)	0.70* (0.57-0.86)	1.10* (1.01-1.21)	0.81 (0.46-1.49)
Facility location	Metro	Reference	Reference	Reference	Reference	Reference
	Urban	1.39 (1.20-1.61)	0.92 (0.85-1.01)	0.98 (0.85-1.14)	0.97 (0.88-1.05)	0.81 (0.42-1.45)
	Rural	1.83* (1.29-2.52)	0.90* (0.65-0.98)	1.05 (0.70-1.55)	0.98 (0.80-1.20)	0.47 (0.0.03-2.23)

OR > 1 is associated with nonadherence to guideline-concordant therapy; OR < 1 is associated with receipt of guideline-concordant therapy.

*Abbreviations:* CI = confidence interval; CoC = commission on cancer; K = thousand; N/A = not applicable; OR = odds ratio.

⁎ *P* < .05.

For patients with rectal cancer, older age (OR, 1.01; 95% CI, 1.01-1.02; *P* < .0001), black race (OR, 1.12; 95% CI, 1.01-1.25; *P* = .04), and treatment at either a community cancer program (OR, 1.27; 95% CI, 1.12-1.43; *P* < .0001) or a comprehensive community cancer program (1.23; 95% CI, 1.14-1.32; *P* < .0001) were associated with nonadherence to guideline-concordant treatment, while having a high income (OR, 0.90; 95% CI, 0.82-0.99; *P* = .03), receiving treatment at more than 1 facility (OR, 0.74; 95% CI, 0.69-0.79; *P* < .0001), receiving treatment at a high-volume facility (OR, 0.70; 95% CI, 0.64-0.77; *P* < .0001) or at a rural treatment center (OR, 0.90; 95% CI, 0.65-0.98; *P* = .03) decreased the likelihood of nonadherence to guideline-concordant treatment. For cervical cancer, Charlson-Deyo comorbidity scores of 1 (OR, 1.22; 95% CI, 1.06-1.41; *P* = .01), having private insurance (OR, 1.88; 95% CI, 1.55-2.30; *P* < .0001) or Medicare (OR, 1.62; 95% CI, 1.29-2.06; *P* < .0001), treatment at a comprehensive community cancer program (OR, 1.61; 95% CI, 1.43-1.80; *P* < .0001) or an integrated network cancer program (OR, 1.30; 95% CI, 1.10-1.54; *P* < .0001), were all associated with nonadherence, while treatment at a high-volume facility (OR, 0.70; 95% CI, 0.57-0.86; *P* < .0001) was associated with decreased likelihood of nonadherence to guideline-concordant treatment.

For patients with lung cancer, Charlson-Deyo comorbidity scores of 1 (OR, 1.32; 95% CI, 1.24-1.41; *P* < .0001 and OR, 1.34; 95% CI, 1.21-1.49 for Charlson-Deyo comorbidity scores of 2 or more, *P* < .0001), higher income, and having private insurance (OR, 1.87; 95% CI, 1.57-2.26; *P* < .0001) or Medicare (OR, 1.67; 95% CI, 1.39-2.03; *P* < .0001) were associated with nonadherence to guideline-concordant treatment, while black race (OR, 0.64; 95% CI, 0.57-0.71; *P* < .0001), younger age (OR, 0.98; 95% CI, 0.98-0.98; *P* < .0001), and treatment at any nonacademic facility were all associated with decreased likelihood of nonadherence (Table 2). For nasopharynx cancer, other race (OR, 0.30; 95% CI, 0.15-0.55; *P* < .0001), treatment at a comprehensive community cancer program (OR, 0.49; 95% CI, 0.31-0.75; *P* = .002) or at an integrated network cancer program (OR, 0.23; 95% CI, 0.08-0.53; *P* = .002) were all associated with receiving guideline-concordant treatment. There were no factors associated with nonadherence to guideline-concordant treatment.

Fig 1 shows the treatments received by the patients in the nonguideline-concordant treatment group in greater detail. For cervical cancer, patients in the nonguideline-concordant treatment group received radical surgery followed by chemotherapy and radiation (24.8%). For rectal cancer, the plurality of patients in the nonguideline-concordant treatment group received adjuvant radiation and multiagent chemotherapy (43.4%). For anal cancer, the plurality of patients in the nonguideline-concordant treatment group were treated with radical surgery followed by chemotherapy and radiation (36.4%). For nasopharynx cancer, most patients in the nonguideline-concordant treatment arm did not receive any therapy (32.2%). For NSCLC, the most common nonguideline-concordant treatment was radical surgery followed by chemotherapy and radiation (31.7%) or radical surgery alone (24.2%).

## Discussion

This is the first study to examine adherence to multimodality standard of cancer care, through the use of a national cancer registry, including a variety of cancer sites. Other studies have included patients with a single cancer site and smaller patient numbers.^6^, ^7^, ^8^, ^9^^,^^19^, ^20^, ^21^, ^22^, ^23^, ^24^ Our analysis shows that 9% of the studied patients with cancer, including 21% of cervical, 3% of nasopharyngeal, 10% of rectal, 7% of NSCLC, and 5% of patients with anal cancer, are receiving nonguideline- concordant cancer treatment. In general, older patients, those with medical comorbidities, and those treated at low-volume facilities were more likely to receive treatment that was not guideline-concordant. The remaining characteristics associated with nonadherence to guideline-concordant treatment were inconsistent and differed across cancer sites. Our findings are in keeping with the reported literature, showing a wide range of adherence with guideline-concordant care based on disease and patient characteristics, both nationally^7^, ^8^, ^9^^,^^20^^,^^22^ and abroad.^25^, ^26^, ^27^, ^28^

Treatment recommendations outlined by the NCCN for cancers included in this analysis are based on high level data showing improved outcomes for patients with cancer with respect to both survival and quality of life. For anal cancer, radiation and chemotherapy have replaced radical surgery as definitive therapy based on data from a number of cooperative group trials showing equivalent survival without the morbidity associated with surgery.^29^, ^30^, ^31^ Although definitive chemotherapy and radiation have long been the standard therapy for anal cancer, 5% of patients are still being treated with radical surgery requiring permanent colostomy. After publication of the practice-changing German rectal trial, neoadjuvant radiation therapy replaced adjuvant radiation as the recommended therapy for locally advanced rectal cancer due to superior locoregional control and colostomy-free survival.^18^ Yet, our study shows that 10% of patients with locally advanced rectal cancer are undergoing surgery first.

National guidelines recommend that patients with locoregionally advanced cervical cancer receive definitive chemotherapy and radiation, rather than upfront surgery. This recommendation derives from data showing that surgery alone is not curative, and treatment with surgery followed by adjuvant therapy results in excessive morbidity.^32^, ^33^, ^34^ Further, brachytherapy is a key component of curative therapy for these patients and must be included as a part of treatment, as reflected in the NCCN guidelines.^16^^,^^35^^,^^36^ Surgery is similarly not recommended for patients with locoregionally advanced nasopharynx cancer because of the inaccessible location of the nasopharynx and its proximity to critical neurovascular structures, making radiation the recommended therapy.^17^^,^^37^ For patients with locally advanced NSCLC, the recommended treatment is concurrent chemotherapy and radiation rather than upfront surgery given the lack of randomized data supporting curative surgery alone for these patients.^4^^,^^38^

Our study shows that there is marked variation in patient factors predicting for nonadherence to practice guidelines across cancer sites. Similar to the analysis done by Bristow et al^8^ for patients with ovarian cancer, we found that patients receiving treatment at high-volume centers were more likely to receive guideline-concordant treatment. For the majority of cancers studied, older age was associated with nonadherence to guideline-concordant treatment, in keeping with data from other studies.^7^^,^^39^, ^40^, ^41^ For example, Wockel et al^7^ showed that older age was associated with nonadherence to guidelines in patients with breast cancer. Although we found that generally older, sicker patients did not undergo guideline-concordant treatment, it is not necessarily due to their inability to tolerate it, given that the nonguideline-concordant treatment was generally more aggressive. For cancers of the anus, lung, and nasopharynx, treatment at academic centers was associated with lower rates of adherence to guideline concordance. One explanation for this finding may be that complex or higher acuity cases may be preferentially referred to academic centers and these cases may have contraindications precluding guideline-concordant treatment. There was great variability among the other factors in terms of predicting for adherence to guidelines, indicating that there is a need for general awareness that some patients are not being treated in accordance with evidence-based medicine, which may adversely affect patient outcomes.

We also found that there was variability in terms of primary site and receipt of guideline-concordant care. Twenty-one percent of patients with cervical cancer are receiving nonguideline- concordant therapy, versus only 3% with nasopharyngeal cancer. The relatively large number of patients with locally advanced cervical cancer receiving nonguideline-concordant treatment may be explained by the fact that there is no seminal paper establishing chemotherapy and radiation as the standard treatment for these patients. The current treatment paradigm for patients with cervical cancer was established over time as more data showed that surgery alone was not curative for these patients and trimodality therapy was unnecessarily morbid.^32^, ^33^, ^34^, ^35^

There are several reasons why providers may deviate from guideline-concordant care when treating patients with cancer. Some may be valid, including contra-indications to delivering the recommended care or patient preference.^42^ However, there are other cases, such as lack of familiarity with treatment guidelines, where modifications to treatment patterns may not be warranted. For example, studies have shown that lack of familiarity with treatment guidelines can result in nonadherence.^25^^,^^43^^,^^44^ Other reasons cited for nonadherence include provider attitudes, such as lack of agreement with the treatment recommendations, or a failure to modify practice after guidelines have been updated with newer recommendations.^44^ Further investigation into the reasons for nonadherence is needed so that invalid causes of treatment nonadherence can be rectified to improve patient care.

Adherence to treatment guidelines can improve the outcomes of patients with cancer. Bristow et al^8^ showed that nonadherence to the standard treatment for patients with ovarian cancer was associated with decreased cancer-specific survival (hazard ratio [HR], 1.33; 95% CI, 1.26-1.41) and suggest that adherence to guidelines may be a useful measure of quality of cancer care provided. Wockel et al^7^ showed that patients with breast cancer not treated according to guidelines had worse recurrence free (HR, 2.10; 95% CI, 1.49-2.98) and overall survival (HR, 2.85; 95% CI, 1.98-4.12). A systematic review and meta-analysis again showed that survival outcomes were improved in patients with breast cancer treated according to guidelines. Others have shown that guideline nonadherence was independently associated with increased cancer-specific mortality for patients with cervical cancer (HR, 1.55; 95% CI, 1.34-1.80),^45^ anal cancer (HR, 1.87; 95% CI, 1.66-2.12),^24^ and nasopharyngeal cancer (HR, 1.46; 95% CI, 1.25-1.69).^23^

Treatment in accordance with national guidelines can also can reduce health care spending. In their retrospective review of Medicare claims data from 2012 to 2015, Williams et al^46^ found that 1 in 6 patients with early-stage breast cancer did not receive guideline-concordant treatment. Guideline-discordant therapy resulted in an additional $936 in monthly costs per patient due to increased emergency room visits and hospitalizations. This increased rate of health care utilization also strains the health care system. Just as importantly, we have established treatments with high quality data showing improvements in patient survival and quality of life, which are not being provided to approximately 1 in 10 patients with cancer, as shown in our study.

There are a number of limitations to this study that should be noted. As with all large cancer registries, the potential for misclassification due to coding errors is 1 such limitation. Incomplete patient information, specifically with respect to patient stage, was another limitation that resulted in the exclusion of many patients from this study. Further, the NCDB does not list specific patient comorbidities, which may have precluded guideline-concordant treatment in certain patients. Although we controlled for Charlson-Deyo comorbidity score, we were unable to identify specific comorbidities known to providers that may have affected care decisions. Additionally, we do not know the reasons why patients did not receive guideline-concordant treatment. In some instances, patients may have refused the recommended therapy or may not have been able to receive the treatment due to other comorbidities or other valid clinical reasons. It should be noted, however, that for the sites we studied, radiation was the recommended therapy while surgery was the nonguideline-concordant treatment. We would not expect patients to be too sick to undergo radiation and to instead receive surgery. In addition, certain patients may have had contraindications to receiving radiation therapy that precluded them from receiving guideline-concordant care, such as inflammatory bowel disease, pregnancy, scleroderma, or other connective tissue disorders.

## Conclusions

This study shows that about 9% of patients with cancer are not receiving cancer therapy according to recommendations made by national guidelines. These patients’ survival may be affected as a result, which should be investigated with future research. In addition, reasons for nonadherence to guidelines should be further investigated so that these root causes may be addressed, in particular for primary sites with a disproportionately large percentage of patients receiving nonconcordant care, such as patients with cervical cancer. As policymakers and payers increasingly move toward a value-based payment model, treatment in accordance with national guidelines should be emphasized. More effort is needed to ensure that all patients are receiving the highest level of care in accordance with evidence-based guidelines.

## Appendix Supplementary materials

Supplementary Table 1: Definition of guideline-concordant and non-guideline concordant therapy, recommended radiation and chemotherapy regimens, and data in support of these recommendations. Supplementary Figures 1-5: CONSORT diagram illustrating included and excluded patients by disease site. Image, application 1

## Footnotes

Supplementary material associated with this article can be found, in the online version, at doi:10.1016/j.adro.2022.100938.
