# Supplementary material for: Nonadherence to Multimodality Cancer Treatment Guidelines in the United States
Source: Adv Radiat Oncol. 2022 Mar 8;7(5):100938. doi: 10.1016/j.adro.2022.100938 (PMC9034283; doi:10.1016/j.adro.2022.100938)

Supplementary Table 1: Definition of guideline-concordant and non-guideline concordant therapy, recommended radiation and chemotherapy regimens, and data in support of these recommendations.

| **Cancer Sub-site** | **Stage** | **Guideline-Concordant Therapy** | **Non-Guideline Concordant Therapy** | **RT Dose** | **Chemo** | **Supporting Data (Ref)** |
| --- | --- | --- | --- | --- | --- | --- |
| Anus | I-III | RT + CT | APR | 45 – 59.4 Gy | MMC/  5Fu | ECOG-7283 (22);  RTOG-8314 (23);  French Trial (24) |
| Rectum | II-III | RT +/- CT 🡪 Surgery | Surgery 🡪 (C)RT | 25 Gy / 5 Fx or  45 – 50.4 Gy | Capecitabine or 5Fu (for long course) | German Rectal Trial (18) |
| Cervix | IIB, III, IVA | RT (including BT) + CT | Hysterectomy | 40 – 50 Gy + 30 – 40 Gy BT boost | Cisplatin | Yessaian et al (25); Yamashita et al (26); Peters et al (27); Viswanthan et al (28) |
| Nasopharynx | I-III | RT | Pharyngectomy | 70 Gy | Cisplatin (for > T1N0 tumors) | Chua et al (30) |
| Lung (Non-small cell) | IIIB | RT | Lobectomy/ Pneumonectomy | 60-70 Gy | Multiple regimens | N/A* |

Abbreviations: RT, radiation therapy; CT, chemotherapy; BT, brachytherapy; Gy, Gray; Fx, fraction; MMC, mitomycin C; 5Fu, 5-fluorouracil

*There are no prospective trials comparing upfront surgery to chemotherapy and radiation for stage IIIB lung cancer patients. Trials comparing induction therapy followed by surgery to chemotherapy and radiation alone for patients with early stage III (ie. Stage IIIA) lung cancer, have failed to show a benefit to surgery after chemotherapy and radiation. Extrapolating from these data, surgery is thus not recommended as upfront treatment for patients with more advanced stage III disease (ie. Stage IIIB)


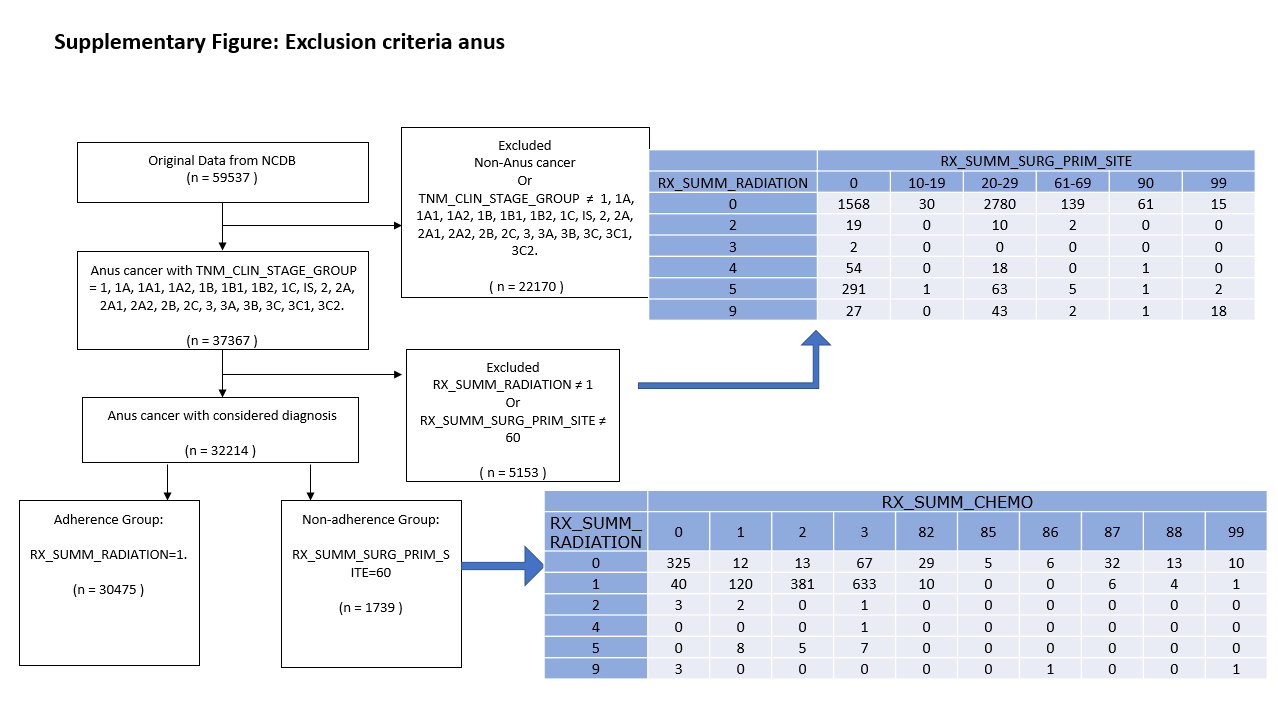


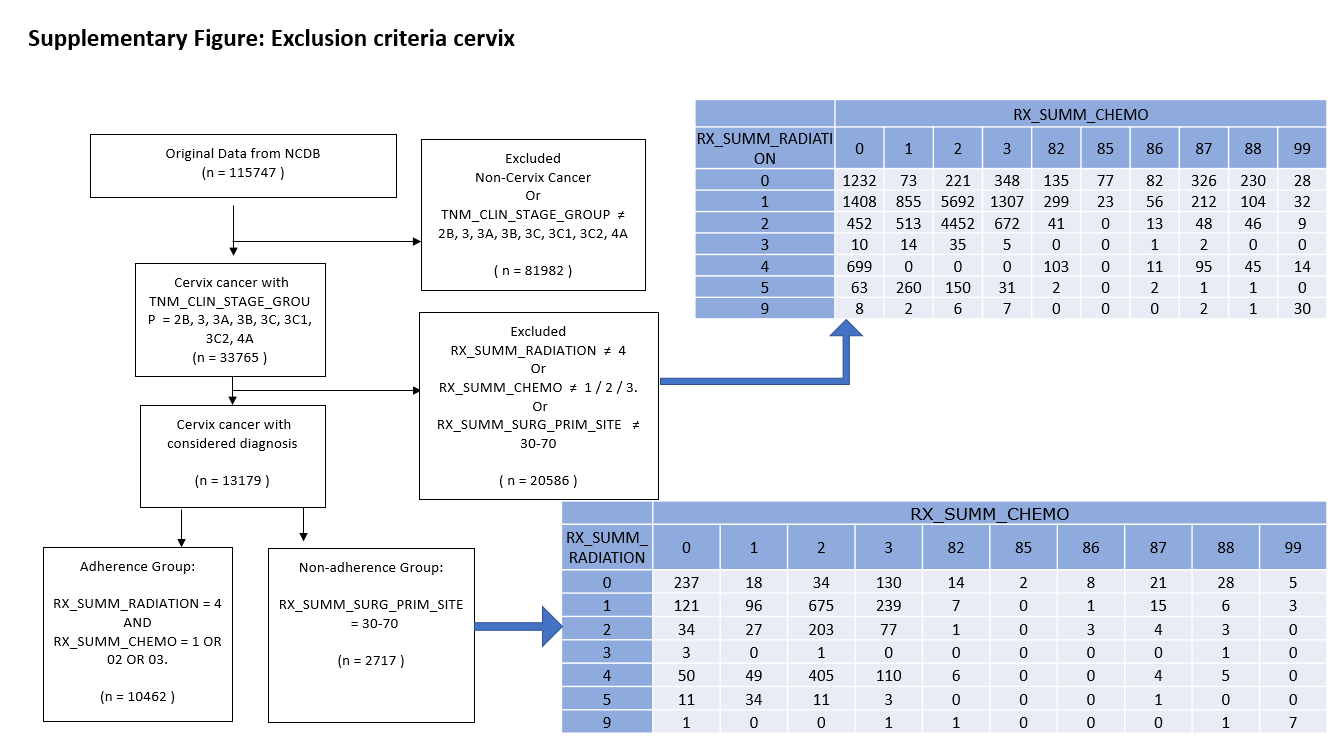


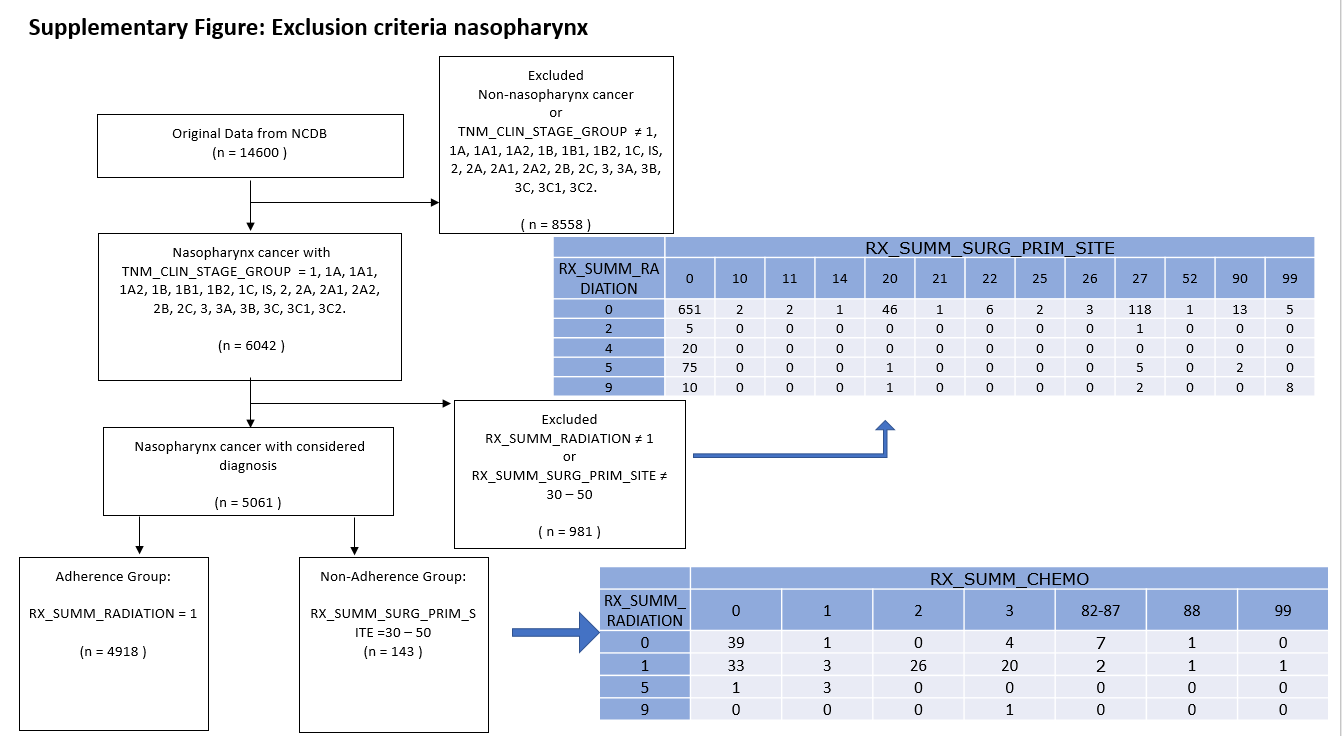


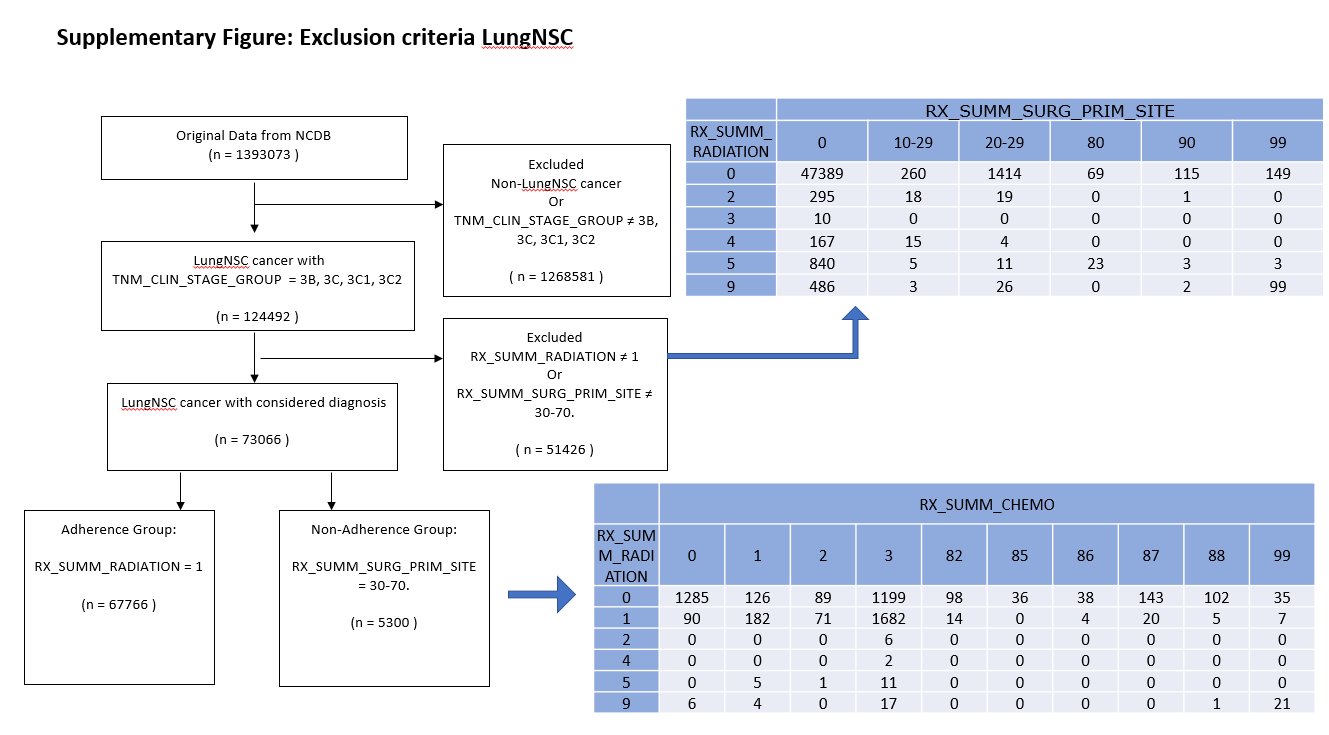


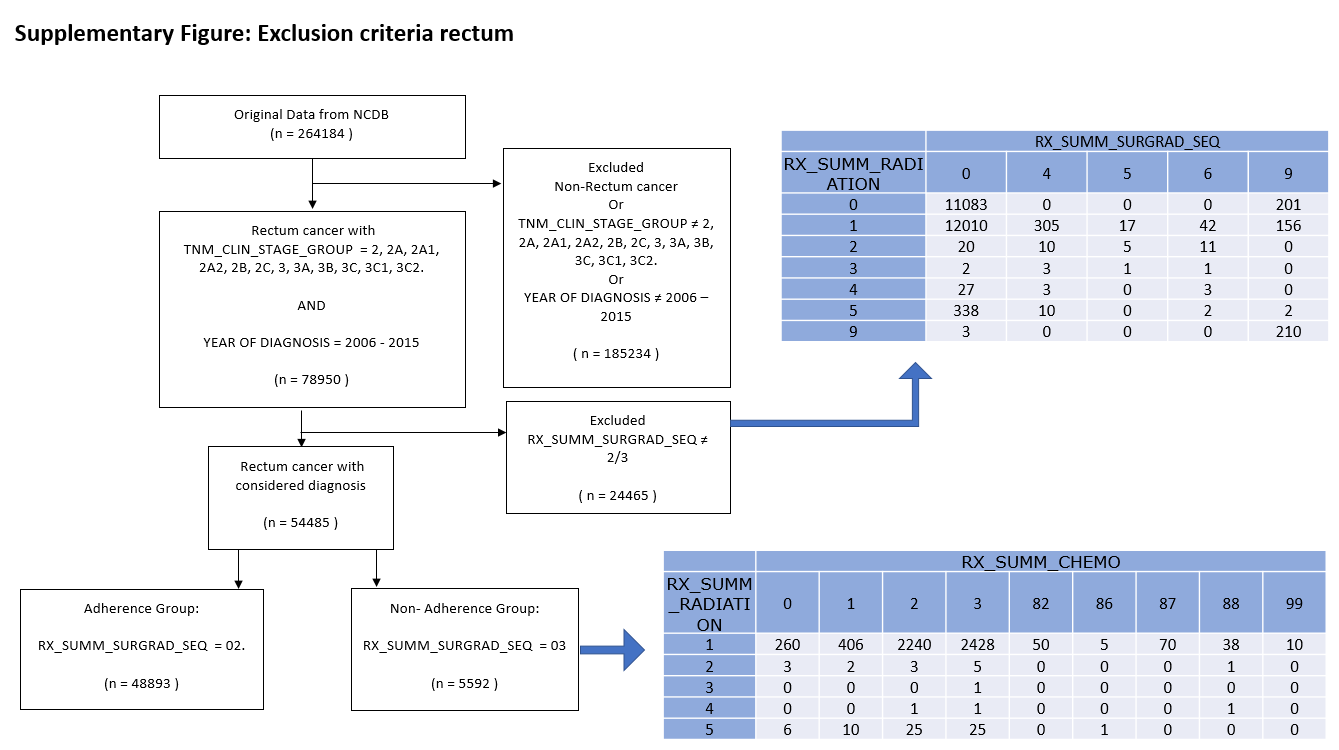

Supplement: Supplementary file 1 — Supplementary Table 1: Definition of guideline-concordant and non-guideline concordant therapy, recommended radiation and chemotherapy regimens, and data in support of these recommendations. Supplementary Figures 1-5: CONSORT diagram illustrating included and excluded patients by disease site. [file mmc1.docx]
